# Assessment of Perception and Barriers toward Orthodontic Treatment Needs in the Saudi Arabian Adult Population

**DOI:** 10.3390/healthcare10122488

**Published:** 2022-12-09

**Authors:** Abdulrahman K. Alshammari, Ammar A. Siddiqui, Nawaf H. Al Shammary, Yasser R. Malik, Mohammad Khursheed Alam

**Affiliations:** 1 Department of Preventive Dentistry, College of Dentistry, University of Ha’il, P.O. Box 2440, Ha’il 81481, Saudi Arabia; 2 Department of Preventive Dentistry, College of Dentistry, Jouf University, Sakaka 72345, Saudi Arabia; 3 Department of Dental Research Cell, Saveetha Dental College and Hospitals, Saveetha Institute of Medical and Technical Sciences, Chennai 600077, India; 4 Department of Public Health, Faculty of Allied Health Sciences, Daffodil International University, Dhaka 1341, Bangladesh

**Keywords:** orthodontic, orthodontic treatment, perception, survey, adults, barrier, protrusion

## Abstract

Patients’ decisions regarding orthodontic treatment are influenced by a variety of factors, such as their subjective opinions of how their oral cavity looks, age, gender, educational level, and income. The present study aimed to measure the orthodontic treatments needs for the Saudi Arabian population, and also to report potential barriers towards it. It is an observational study with cross sectional design. Data was collected from nationals and residents living in Saudi Arabia and who are at least 18 years old. A total of 1184 adult patients (880 females and 304 males) were included in this study. The population for the study was reached and chosen using a non-probability snowball sampling method. A validated and reliable questionnaire was distributed to the participants electronically through google form. Inferential statistics was applied using Pearson Chi Square test. *p* value < 0.05 was considered significant. The majority of the sample (73.2%) exhibited a favorable attitude toward orthodontic treatment. Most participants including male and female gender wanted orthodontic treatment primarily to have their protruding teeth fixed. The proportion of persons who cited chewing difficulty as the reason they needed orthodontic treatment was strongly influenced by the monthly income variable (*p* = 0.005). Cost and time of orthodontic therapy were the biggest obstacles to undergoing treatment, accounting for 34.5% and 19.5%, respectively. There was highly significant difference between the underlying medical conditions and the age and monthly income variables (*p* = 0.000). Based on the findings of the present study, it can be concluded that protruding teeth are the most important treatment needs perceived by the Saudi population, followed by spacing in between teeth, crowding of teeth, and unaesthetic smile. Cost of the orthodontic treatment was identified as the most significant barrier to the treatment needs, followed by long duration of orthodontic treatment, and pain.

## 1. Introduction

Patients seeking orthodontic treatment are chosen based on both objective and subjective criteria. While objective orthodontic treatment need is determined by specialists’ clinical findings, subjective orthodontic treatment need is determined by a few factors, one of which is the patient’s self-perception of orthodontic treatment need [1].

Identifying and measuring the treatment needs of a community is critical when planning orthodontic treatment [2]. The practical and emotional benefits of orthodontic treatment have yet to be proven conclusively in the dentistry literature [3]. Individuals with orthodontic issues may experience psychological and social effects that contribute to their mental health, which have been overlooked in earlier decades. The overall appearance of one’s teeth and face can have an impact on one’s mental image of one’s body, which can lead to personal happiness [4]. The absence of detrimental effects of oral conditions on social life and a positive sense of dentofacial self-confidence have been characterized as oral health-related quality of life (OHRQoL). Patient personality factors, such as internal control and self-perception competency, may influence the development of psychosocial impact [5]. Patient compliance and, as a result, treatment outcomes are heavily influenced by the patient’s attitude toward orthodontics. As a result, orthodontists must first understand the mindset of their patients, as well as their understanding of their dental issues and solutions, to ensure that both treatment planning and treatment can take place without hindrance [6].

At different ages, patients’ expectations for orthodontic treatment differ. Adult orthodontic therapy has traditionally been used to mask the treatment of adult patients in their 20s and early 30s, rather than teenagers [7]. This concept of “adult patients” has been applied to middle-aged and elderly patients globally. The shift in perception is due to the profession’s increased ability to manage problems and patients’ desire to keep their natural teeth and improve their function as well as attractiveness [8]. Now the issue is, what is the most common orthodontic treatment requirement and what is the most prevalent orthodontic ailment that has multiple consequences in a person’s life?

Due to its deep implications on the psychological and practical aspects of life, malocclusion can have a substantial impact on an individual’s quality of life [9]. Basic functions such as eating, and speaking might be difficult for people who have a significant malocclusion [10]. The condition of one’s teeth can have a considerable impact on one’s emotional, psychological, and social well-being [11]. Malocclusion is now ranked with other oral health issues including tooth caries and periodontal disease [12]. A research of 1459 children aged 9 to 17 undertaken in Saudi Arabia, by Abdellatif and Al-Emran discovered that the majority of subjects (92%) believe that proper occlusion is very important [13]. Patients’ subjective impressions of their oral look, age, gender, peer-group standards, and self-esteem all play a role in their decision to undertake orthodontic treatment [14]. To the best of our knowledge this is the first study of its kind of for Saudi Arabian population that measured the orthodontic treatments needs of the adult Saudi population, and also accounts for potential barriers towards it.

## 2. Material and Methods

### 2.1. Study Design

This was an epidemiological observational exploratory study that used a cross sectional survey.

### 2.2. Study Setting

The present study collected data electronically using a Google documents form. Participants were recruited by asking them to fill out an online form that was distributed over social media. Additionally, participants were requested to share the link with people they may know. Various social media communication applications such as twitter, Facebook and WhatsApp was used to collect the information. Data was collected from every region of Saudi Arabia. Participants provided informed consent. Data for the present study were collected from 20 April 2022 to 22 May 2022.

### 2.3. Study Participants

In total, data was gathered from *N* = 1184 respondents from Saudi Arabia, using a non-probability snow ball sampling technique. However orthodontic treatment need, and barriers to treatments was answered by *N* = 867 participants, who had a previous experience of going to an orthodontist. The inclusion criteria were adults 18 years or above, ability to read and understand the questionnaire, provision of informed consent for participation, residency in Saudi Arabia. The exclusion criteria include participants below 18 years of age, and those with significant medical problems. The ethical approval for the present study was applied and approved through a scientific research committee at the deanship of research, University of Hail, Saudi Arabia, approval number (H-2022-033).

### 2.4. Study Variables

The present study contained sociodemographic variables such as age, sex, nationality, region of resident in Saudi Arabia, and level of education and monthly income. The second part of the questionnaire included 14 items for orthodontic treatment needs (teeth protrusion, crowding, spacing, jaw prognathism, asymmetry, teeth malalignment, teeth tipping, dentist recommendation, friends/family receiving treatment, TMJ disorder, hard chewing, occlusal problems unaesthetic smile and fashion). The third part of the questionnaire included 13 barriers toward orthodontic treatment (treatment cost, pain, braces draw attention, feeling too old for orthodontic treatment, more loss than gain, no time to attend orthodontic visits, underlying medical history, peer advice, TMJ problems, teeth do not need treatment, long treatment duration, long waiting list, and periodontal complication). Study participants were allowed to select a maximum of 3 items for the orthodontic treatment needs and probable barriers to the treatment based on their perceptions.

### 2.5. Data Collecting Tool

An existing, validated, and reliable questionnaire was used to collect data from previous study [2]. Through the use of forward and reverse translations, the questionnaire was translated into Arabic. An Arabic-speaking bilingual specialist first translated the questionnaires from English to Arabic. Then, it was decided to approach a bilingual expert in Arabic to find and correct inconsistencies between the forward translation and the current version. After that, a second translator independently translated the survey back into English. A satisfactory version of the questionnaire was reached after addressing and reviewing any potential differences. The questionnaire underwent a pilot test with 15 laypeople in order to assess its reliability, clarity, and time requirements. Using the findings of the pilot test, the questionnaire was improved and modified. The analysis excluded the responses from consideration.

### 2.6. Statistical Analysis

The present study had categorical data. Descriptively, it was displayed as number and percentages. Inferential statistics was applied using Pearson Chi Square test. *p* value < 0.05 was considered significant. Data was coded and recorded in a Microsoft excel file, and later was transferred to the Social Sciences Statistical package version 22 for analysis.

## 3. Results

This study included 1184 participants, with females accounting for (74.3%) of the respondents and males comprising the remaining. In terms of age groups, 663 participants were aged between 20 and 29, which accounted for 56% of the participants. The next highest age group were those individuals aged between 30 and 39, and the least well represented group were patients aged over 60 years. From a location perspective, most responses came from the central region, representing (62.8%) participants; the west and east were represented by 12.2% and 17% respectively. The majority of respondents (66.4%) held bachelor’s degrees, while postgraduates totaled 10.6%. Finally, the majority of the sample demonstrated an interest in orthodontic treatment: 73.2% were in favor compared to 26.8% who were not. See Table 1 for more information.

For many individuals (18%), protruding teeth motivated them to seek orthodontic treatment, while 13.2% cited teeth spaces as their main motivator. Next came an unaesthetic smile followed by temporomandibular disorder (TMD). For most males and females, the primary reason for seeking orthodontic treatment was to fix their protruding teeth. The second-most prevalent reason among the genders diverge: females cited teeth spacing, while for males, the reason was teeth crowding. From an age perspective, respondents in the 20–29 age group were most concerned about protruding teeth, followed by spacing and unaesthetic smiles. People in the subsequent age group (i.e., aged 30 to 39) voiced similar concerns, except that crowding replaced the smile issue. For people in the 40–49 age group, protrusion and spacing comprised their primary concerns, followed by crowding and an unaesthetic smile. See Table 2 for further information.

According to the findings, no significant difference (*p* > 0.05) existed between people’s earnings, location, nationality, education level, age, and gender in terms of protruding teeth driving their desire to receive orthodontic treatment. However, a notable connection (*p* = 0.045) was found between gender and teeth crowding; the majority of males in the sample cited teeth crowding as their main reason for seeking treatment. People’s location revealed a noteworthy finding, in the sense that a significant difference (*p* = 0.018) existed between this variable and teeth spacing. However, none of the included variables (*p* > 0.05) indicated a noticeable difference concerning post-extraction teeth tipping, dentists suggesting getting treatment, a friend or family undergoing similar procedures, TMJ disorder, occlusion, and unaesthetic smiles. The earnings variable showed a noticeable difference (*p* = 0.002) in terms of people citing hard chewing as their driver to seek orthodontic support: more people with lower earnings sought this kind of care. Seeking orthodontic care as an aesthetic or fashion-based choice seemed only to have a noteworthy difference (*p* = 0.003) in relation to the age variable. See Table 3 for more information.

The study found that financial concerns were the most prevalent reason for not getting orthodontic treatment (34.5%.) The next most-cited reason was the length of treatment (19.5%), followed by pain and discomfort (15.7%). Financial constraints were the main reason for both males and females not to pursue orthodontic care. The next most-cited reason for both genders was the length of treatment, followed by painful treatment. For males, the least-cited obstacle was TMJ disorder, while for females, it was underlying medical issues. In terms of age, the findings showed a similar pattern: the cost was the most prohibitive factor, followed by the length of treatment and, in third place, the pain when receiving treatment. See Table 4 for more information.

In terms of the cost of treatment and gender, the findings showed significant difference (*p* = 0.047). Fewer males cited cost as an obstacle to receiving treatment than their female counterparts. No significant difference (*p* > 0.05) existed between the level of discomfort associated with orthodontic treatment and individuals’ earnings, location, education level, age, and gender, although it should also be noted that feeling too old for treatment only indicated a significant difference (*p* = 0.013) in relation to the gender variable.

According to the findings regarding underlying medical conditions, a significant difference (*p* = 0.000) only revealed itself concerning the age and earnings variables. Many variables failed to show a significant difference (*p* > 0.05) when applied to the length of treatment, concerns over waiting lists and periodontal problems. However, the link between location and the length of treatment was significant (*p* = 0.018), as was the relationship between age and periodontal complication (*p* = 0.034). See Table 5 for more information.

## 4. Discussion

The purpose of this study was to assess the Saudi Arabian population’s need for orthodontic care and to identify any potential obstacles. The majority of both male and female participants in the study sought orthodontic treatment primarily to have their protruding teeth fixed. Teeth malocclusion can have different detrimental effects, including compromising occlusal harmony. Consequently, it should be noted that teeth malocclusion potentially harms a person’s oral health, self-perception, capacity to function, and social and psychological well-being [15,16,17]. Incidences of malocclusion vary according to age. For instance, in Saudi Arabia, a study of children and adolescents found that between 21% and 55.4% of participants showed signs of malocclusion [18,19,20,21]. According to Livas [22] (2013), most such studies have focused on youngsters, although dental issues do not exist solely in this demographic; more adults are seeking orthodontic treatment as well, due partly to the increased focus on smile aesthetics and being accepted socially. These are representative findings because most people in Saudi Arabia fall into the 25 to 54 age group. Of equal importance is understanding people’s attitudes towards orthodontic treatment and any obstacles that may hinder such treatment. This information can help healthcare providers develop and implement relevant policies; since no research has focused on the adult population and their perceptions of orthodontic treatment, this study can provide viable supplemental data.

In this study’s sample, 58.1% of individuals with a high interest in receiving orthodontic treatment were over 40, which aligns with the study by Kim [2]. Research further revealed that younger and older adults positively responded to the idea of orthodontic treatment than in a previous study [2]. For example, in this study, patients in their 40s and 50s accounted for 60.8% and 53.7%, respectively, while younger patients who were enthusiastic about orthodontic treatment amounted to 82.1%.

This study also found a noticeable difference between male and female participants because the latter outnumbered the former by 75.3% to 67.1% in terms of high response levels to orthodontic care. Such a finding matches previous research [2,23,24,25]. However, both genders show increased interest in treatment and could clarify why more older patients want orthodontic care.

There are different reasons for a person wanting to receive treatment, but there is always one reason that is more pressing than others. Thus, this study has attempted to clarify and prioritize these reasons based on information provided by various age groups. Results have found that the reasons for treatment tend to comprise protruding lips, spacing, and unaesthetic smiles. More focused studies have found that for people in their 20s and 30s, protrusion and spacing account for the most and second-most prevalent complaints, respectively. However, these two age groups then diverge: the third most-common complaint for the 20–29 age group was an unaesthetic smile while for those in their 30s, it was crowding. Crowding and spacing may result from the decreased support offered by a compromised periodontium [26,27]. The primary complaint mentioned by these two age groups mirrored a previous study, which found that protrusion topped the list of reasons for wanting orthodontic care. However, this study differed from previous research, which found that crowding and asymmetry comprised the next most common issue, followed by asymmetry and crowding [2].

This study found that people of the same age in different places had contrasting reasons for wanting orthodontic care, which may illustrate how cultural norms differ or that fears over compromised oral hygiene may prompt older patients’ concerns over malocclusion. Equally, adult patients aged 40 to 49 cited their primary reasons for seeking treatment as protrusion and spacing, crowding, and an unaesthetic smile. Meanwhile, people aged 50 to 59 cited spacing as their primary reason, followed in second place by an unaesthetic smile and in third place by protrusion and crowding. Unlike other research, such as Kim [2], this study found that an unaesthetic smile ranked among the top three drivers for patients in their 40s and 50s in terms of seeking orthodontic care. Such a finding could further indicate how a person’s background can influence their decision-making process.

The result of the study showed a significant difference between the genders regarding teeth crowding as a reason for seeking orthodontic treatment. Most of the male participants considered teeth crowding as their primary motivator for seeking orthodontic care. This study found that a notable difference existed between the genders in terms of crowded teeth informing their decision to seek orthodontic care. For instance, teeth crowding ranks as the primary orthodontic concern for male patients, a fact illustrated by research in Saudi Arabia that found that 36.78% of subjects had crowded teeth [28]. Despite such findings, however, a note of caution is advised: such research has focused on children and adolescents. Further research has also revealed that teeth crowding is an issue for more men than women [21].

In terms of participants’ location, a notable difference was revealed between this variable and teeth spacing as the motivator for getting orthodontic care. Conversely, teeth spacing showed no such difference concerning gender, age, education, and earnings, which suggests that individuals from different places have contrasting attitudes about teeth spacing. People’s earnings differed significantly with hard chewing in terms of wanting orthodontic care. In other words, this kind of care was more common for people with lower earnings. Hard chewing is a widespread issue and can occur because of malocclusion; for instance, it can result from teeth becoming tipped post-extraction.

The cost of treatment proved prohibitive for many people and was the primary obstacle to seeking care for 34.5% of respondents in this study. The second-most-common reason for not getting treatment was the length of time it would take, followed by how much discomfort it would cause. These factors were typical amongst all age groups. Moreover, not many patients thought they were too old to receive care, which might clarify why such a large proportion of adults responded positively to the notion of receiving orthodontic care.

The two most notable obstacles to receiving care were the cost and length of time it would take, which matches research conducted in Korea [2]. However, this study found that discomfort and pain comprised the third obstacle, differing from the previous study [2] in which the third obstacle was patients feeling too old for treatment. Moreover, concerns over age differed significantly from gender. Further noteworthy differences existed between underlying medical issues and the age and earnings variables. Such findings suggest that policy-makers should evaluate the cost of orthodontic procedures and the provision of insurance in addition to clarifying that treatment is available to people with systemic conditions as long as the correct precautions are taken.

Although this study has undertaken thorough research, it nevertheless encountered limitations. Due to lack of resources and logistic support, the present study has collected data using a non-probability sampling technique. So, the data may not fully represent its population. Recall bias might also be an issue. Thus, further research encompassing multi-center studies would help to clarify geriatric patients’ requirements and their concerns over receiving orthodontic treatment; an online survey, such as this study, may not have reached such a demographic.

## 5. Conclusions

The vast majority of the sample showed a positive attitude toward orthodontic treatment (73.2%). Most of the male and female participants sought orthodontic treatment primarily to correct their protruding teeth. This study found that the monthly income variable significantly affected the proportion of people who cited difficult chewing as the reason for seeking orthodontic treatment. According to the study, the main deterrent to receiving orthodontic treatment was cost and the length of the orthodontic treatment representing (34.5%) and (19.5%) respectively. The findings addressing the underlying medical issues showed that only the age and wages variables showed a significant effect. These findings imply that policy-makers should assess the cost of orthodontic treatments and the availability of insurance in addition to reiterating that treatment is accessible to those with systemic diseases as long as the proper precautions are taken.

## Figures and Tables

**Table 1:** Demographic Characteristics of the Participants (*n* = 1184).

Variables	Frequency (*n*)	Percentage (%)
**Gender**		
Male	304	25.7
Female	880	74.3
**Age**		
20–29 Years	663	56
30–39 Years	311	26.3
40–49 Years	161	13.6
50–59 Years	41	3.5
60 years and above	8	0.7
**Nationality**		
Saudis	1135	95.9
Non-Saudis	49	4.1
**Area of living**		
Eastern region	201	17
Western region	56	4.7
Northern region	144	12.2
Southern region	40	3.4
Central region	743	62.8
**Education level**		
Primary	8	0.7
Secondary	22	1.9
High School	242	20.4
Bachelor	786	66.4
Postgraduate study	126	10.6
**Monthly Income**		
<1000 Riyals	367	31
1001–5000 Riyals	397	33.5
5001–10,000 Riyals	162	13.7
10,001–15,000 Riyals	136	11.5
15,001–20,000 Riyals	46	3.9
>20,000 Riyals	43	3.6
Ever thought of receiving orthodontic treatment		
Yes	867	73.2
No	317	26.8

**Table 2:** Reasons for seeking orthodontic treatment based on gender and age group.

Variable	Priority in Chief Complaint		
Reason for seeking Orthodontic Treatment	1st	2nd	3rd
	Protrusion (318) 18.0%	Spacing (282) 16.0%	Unesthetic smile (232) 13.2%
Gender	1st	2nd	3rd
Female	Protrusion	Spacing	Unesthetic smile
Male	Protrusion	Crowding	Spacing
Age group (year)	Priority in chief complaint according to age		
	1st	2nd	3rd
20s	Protrusion	Spacing	Unesthetic smile
30s	Protrusion	Spacing	Crowding
40s	Protrusion and spacing	Crowding	Unesthetic smile
50s	Spacing	Unesthetic smile	Protrusion and Crowding

**Table 3:** Relationship between demographic variables and reason for seeking orthodontic treatment.

No.	Reasons for Seeking Orthodontic Treatment	Gender	Age	Level of Education	Nationality	Region	Monthly Income
1	Teeth protrusion	n.s	n.s	n.s	n.s	n.s	n.s
2	Teeth crowding	0.045 *	n.s	n.s	n.s	n.s	n.s
3	Jaw prognathism	n.s	n.s	n.s	n.s	n.s	n.s
4	Asymmetry	n.s	n.s	n.s	n.s	n.s	n.s
5	Teeth malalignment	n.s	n.s	n.s	n.s	n.s	n.s
6	Spacing between teeth	n.s	n.s	n.s	n.s	0.018 *	n.s
7	Tooth tipping after extraction	n.s	n.s	n.s	n.s	n.s	n.s
8	Dentists’ recommendation	n.s	n.s	n.s	n.s	n.s	n.s
9	Friends/family receiving treatment	n.s	n.s	n.s	n.s	n.s	n.s
10	TMJ disorder	n.s	n.s	n.s	n.s	n.s	n.s
11	Hard chewing	n.s	n.s	n.s	n.s	n.s	0.002 *
12	Occlusion problem	n.s	n.s	n.s	n.s	n.s	n.s
13	Unesthetic smile	n.s	n.s	n.s	n.s	n.s	n.s
14	Fashion	n.s	0.003 *	n.s	n.s	n.s	n.s

(n.s = non-signficant. * significant *p*-value).

**Table 4:** Barriers toward orthodontic treatment based on the gender and the age group.

Variable	Priority of Barrier toward Orthodontic Treatment		
**Order of barrier toward orthodontic treatment**	1st	2nd	3rd
	Cost (632) 34.8%	Long treatment duration (355) 19.5%	Pain (285) 15.7%
Gender	1st	2nd	3rd
Female	Cost	Long treatment duration	Pain
Male	Cost	Long treatment duration	Pain
Age group (year)	**Priority in the Barrier toward Orthodontic Treatment**		
	1st	2nd	3rd
20s	Cost	Long treatment duration	Pain
30s	Cost	Long treatment duration	Pain
40s	Cost	Long treatment duration	Pain
50s	Cost	Long treatment duration	Pain

**Table 5:** Relationship between demographic variables and the barriers toward orthodontic treatment.

No.	Barrier toward Orthodontic Treatment	Gender	Age	Level of Education	Nationality	Region	Monthly Income
1	Treatment cost	0.047 *	n.s	n.s	n.s	n.s	n.s
2	Pain	n.s	n.s	n.s	n.s	n.s	n.s
3	Draws attention	n.s	n.s	n.s	n.s	n.s	n.s
4	Age (too old)	0.013 *	n.s	n.s	n.s	n.s	n.s
5	More loss than gain	n.s	n.s	n.s	n.s	0.004	n.s
6	I don’t have time to attend the orthodontic visit						
7	Underlying medical history	n.s	0.000 *	n.s	n.s	n.s	0.000 *
8	Peer advice (Family, friends, dentists, etc.)	n.s	n.s	n.s	n.s	n.s	n.s
9	TMJ problem	n.s	n.s	n.s	n.s	0.039	n.s
10	My teeth are good and does not need treatment						
11	Long treatment duration	n.s	n.s	n.s	0.018	n.s	n.s
12	Long waiting list	n.s	n.s	n.s	n.s	n.s	n.s
13	Periodontal complications	n.s	0.034 *	n.s	n.s	n.s	n.s

(n.s = non-signficant. * significant *p*-value).
